# Scholarly Output in Peruvian National Dentistry according to Gender Disparity: A 10-Year Bibliometric Study

**DOI:** 10.1155/2022/7854479

**Published:** 2022-06-26

**Authors:** Frank Mayta-Tovalino, Josmel Pacheco-Mendoza, Daniel Alvitez-Temoche, Roman Mendoza, Franco Mauricio, John Barja-Ore, Maria Eugenia Guerrero

**Affiliations:** ^1^Vicerrectorado de Investigación, Universidad San Ignacio de Loyola, Lima, Peru; ^2^Unidad de Investigación en Bibliometría, Universidad San Ignacio de Loyola, Lima, Peru; ^3^Academic Department, Faculty of Dentistry, Universidad Nacional Federico Villarreal, Lima, Peru; ^4^Dirección de Investigación, Universidad Privada del Norte, Lima, Peru; ^5^Academic Department of Medical and Surgical Stomatology, Faculty of Dentistry, Universidad Nacional Mayor de San Marcos, Lima, Peru

## Abstract

**Objective:**

To evaluate Peruvian scientific publications in dentistry according to sex disparity (2011-2020).

**Methods:**

This was a retrospective bibliometric study. The unit of analysis was made up of Peruvian dentistry publications indexed in the Scopus database during the last 10 years. Records with metadata (410) corresponding to the period 2011-2020 were downloaded and standardized and refined by analyzing the metadata. The search strategy was developed based on the individual profiles of each Peruvian institution that has a dental school or college. It was evaluated according to the AF-ID of each institution in the Scopus database. In addition, the information provided by the Scopus SciVal tool was used. Finally, publications, impact, and collaboration indicators were used, such as total number per document, per author, average of citations, *h*-index, collaboration rate, number of institutions, the Source Normalized Impact per Paper indicator, the CiteScore, and the Scopus Field-Weighted Citation Impact.

**Results:**

The greatest increase was evident in 2018, with 2019 and 2020 being the maximum peak of scientific publication growth. However, sustained growth has not been evidenced in relation to the female sex. The analysis of coauthorship by the authors revealed four large clusters, of which the first three were represented by male researchers, such as Arriola-Guillen L., Mayta-Tovalino F., and Mendoza-Azpur G., and one by a female, Guerrero María E. Evaluating the national scientific publication in dentistry according to the CiteScore, it was found that most of the publications (145) from Peru were published in Q4 journals, although 90 manuscripts were published in Q1 journals.

**Conclusions:**

The Peruvian national dental publication in the last 10 years was mainly supported by male dentists, which invites us to reflect on the need to equalize opportunities so that female researchers can also reduce these gaps.

## 1. Introduction

In recent years, with technological advances and the development of new materials, remarkable progress has been made in scientific research in the fields of health sciences, including dentistry [1]. With the implementation of the new University Law 30220 of 2015, drastic changes were made, highlighting the importance of research even for undergraduates.

Because of this, there has been a great increase in scientific publications, today being a pillar element in the academic field in the health sciences. Interest in professional research has been reflected in multiple publications in different lines of research [1–3]. The main purpose is to generate new knowledge and apply it in the clinical field to improve the quality of life of patients, either through new, less invasive treatments or through the publication of new and better materials [4–6].

However, the gradual increase in scientific publications must be evaluated considering quality over quantity [7–9]. Therefore, it is very important to recognize the role of women in science, since recently there has been a worldwide increase in the entry and contribution of these professionals to dentistry worldwide. In this way, bibliometric studies allow the evaluation of the quality of publications through indicators and techniques that measure bibliographic data from publication to the scientific productivity of each researcher, university, and scientific association. To avoid potential bias, certain evaluation parameters and indices are used, such as Source Normalized Impact per Paper (SNIP), CiteScore, CiteScore Rank, and Field-Weighted Citation Impact (FWCI), which are based on the calculation of the citation rate in the manuscript [1–3].

Therefore, bibliometric studies represent a valuable means to analyze scientific publications at the demographic level by institutions, researchers, and even trends. In this study, a bibliometric study of the original scientific publications is carried out, focusing on the approach of sex and evaluation of the trend of scientific publication since the implementation of the new University Law.

## 2. Methods

### 2.1. Study Design

The study was a retrospective bibliometric study. The unit of analysis was made up of publications of Peruvian dentistry (microlevel) indexed in the Scopus database during the last 10 years (2011-2020).

### 2.2. Search Strategy

**Figure 1:**
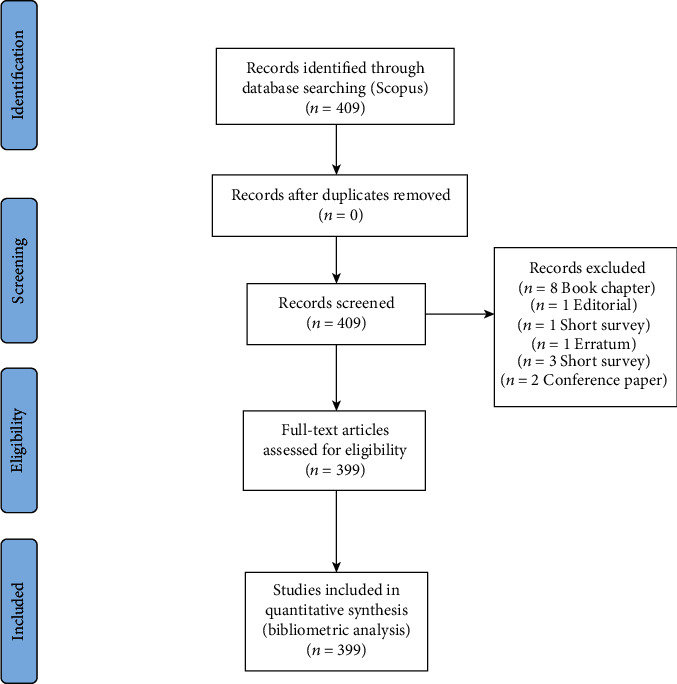
Flow diagram.

On June 7, 2021, 409 manuscripts corresponding to the period 2011-2020 were identified, which were normalized and refined by analyzing the metadata. The publications included article designs (331), review (21), letter (35), book chapter (8), and note (12) among others (Figure 1). The Scopus base was used because it is a source that houses numerous scientific journals. Then, the following formula was used in Scopus: SUBJAREA (dent) AND AFFILCOUNTRY (Peru) AND (LIMIT-TO (PUBYEAR, 2020) OR LIMIT-TO (PUBYEAR, 2019) OR LIMIT-TO (PUBYEAR, 2018) OR LIMIT-TO (PUBYEAR, 2017) OR LIMIT-TO (PUBYEAR, 2016) OR LIMIT-TO (PUBYEAR, 2015) OR LIMIT-TO (PUBYEAR, 2014) OR LIMIT-TO (PUBYEAR, 2013) OR LIMIT-TO (PUBYEAR, 2012) OR LIMIT-TO (PUBYEAR, 2011).

### 2.3. Data Collection

The search strategy was developed based on the individual profiles of each Peruvian institution that has a dental school or faculty. It was evaluated according to the AF-ID of each institution in the Scopus database during the period 2011-2020. In addition, the information provided by the Scopus SciVal tool was used, from which all statistical data corresponding to scientific publication in dentistry in Peru were extracted. For data processing, the SciVal (Elsevier) system was used, which uses four different sections for information analysis, which include generalities, comparative evaluation, collaboration, and trends; within these sections, you can analyze countries, institutions (academics, industry, government, hospitals, and others), authors, publications, subject fields, and journals; and with the Microsoft Excel® 2019, descriptive statistics were performed, calculating frequencies and percentages for each study variable.

### 2.4. Indicators

The SNIP indicator from Scopus was used, which is a metric that compares publications in the same area of knowledge. For its calculation, it counts the frequency of citations and the immediacy of the impact of the citation. In addition, the CiteScore was used, which measures the impact of the publication, which is calculated through the citations received and divided by the total number of documents published in the last 3 years. CiteScore Rank provides the position of the scientific journal within its category. Finally, the FWCI was calculated since it measures the impact of citations weighted by a field of knowledge. It is calculated from the relationship between the total number of citations received by the result of the denominator and the total number of citations that would be expected according to the average of the thematic field. For example, an FWCI of 1.55 would mean 55% more quoted than expected.

### 2.5. Statistical Analysis

Descriptive statistics of the scientific publications of all medical schools evaluated were obtained and tabulated in Excel. Bibliometric analysis was performed using the Scopus SciVal tool. The search strategy was developed and downloaded in CSV (a comma-separated value) format to be later exported to Scopus SciVal, from which all statistical data corresponding to scientific publications in dentistry were extracted.

## 3. Results

### 3.1. Scientific Publications according to Sex Disparity

**Figure 2:**
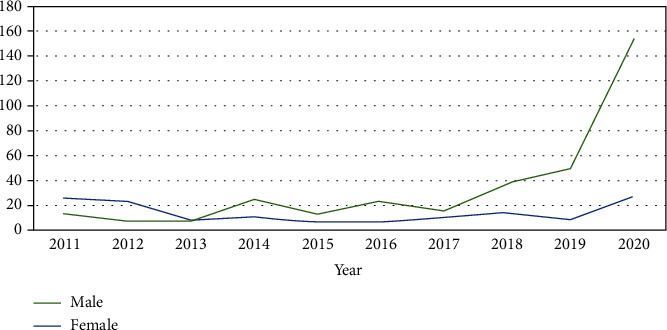
Scientific production of the faculties of dentistry in Peru in Scopus according to gender disparity during 2011-2020.

A notable and constant increase in scientific publications was observed in male dentists. The greatest increase was evident from 2018, with 2019 and 2020 being the maximum peak of scientific publication growth. However, sustained growth has not been evidenced in relation to the female sex (Figure 2).

### 3.2. Coauthorship by the Author

**Figure 3:**
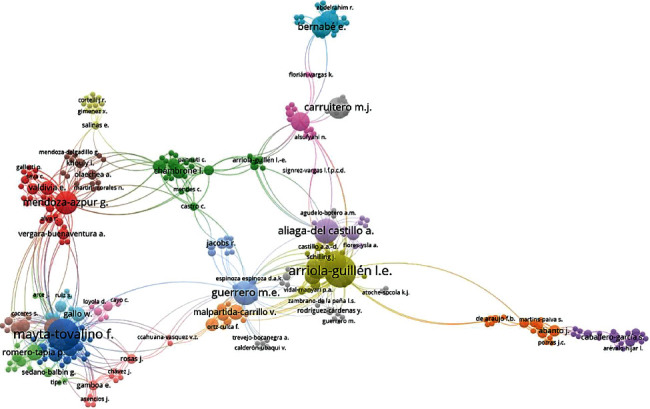
Coauthorship by author.

When conducting the coauthorship analysis by the author, four large clusters were identified, of which the first three were represented by male researchers, such as Arriola-Guillen L., Mayta-Tovalino F., and Mendoza-Azpur G., and one by a female researcher, Guerrero María E. (Figure 3).

### 3.3. Coauthorship by Country

**Figure 4:**
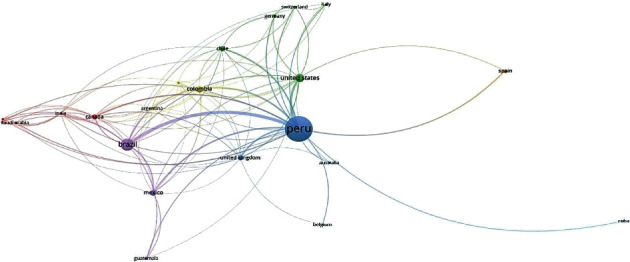
Coauthorship by country.

When conducting the analysis of coauthorship by country, it was found that Peru had different nationalities that contributed to the national scientific publications, with Brazil being the main country with which there is collaboration in coauthorship followed by Colombia, Chile, the United States, and the United Kingdom (Figure 4).

### 3.4. Cooccurrence by Key Words

**Figure 5:**
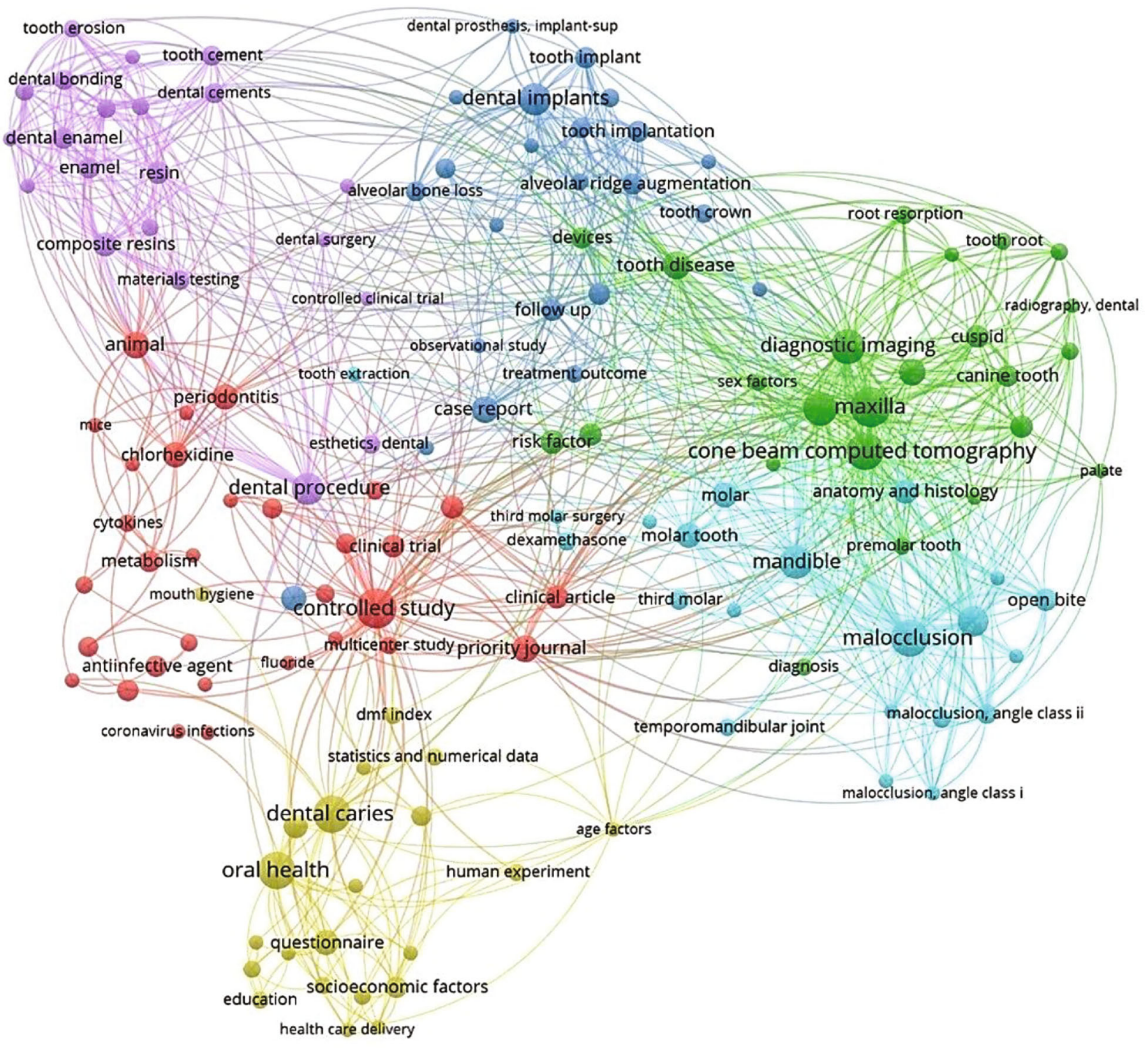
Cooccurrence by key words.

When analyzing the cooccurrence of keywords, five large clusters were evidenced that did not have a clear homogeneity because there were different words that represented the main areas of research in Peruvian dentistry. The words dental caries, cone-beam computed tomography, maxilla, controlled study, periodontics, dental implants, and composite resin are the main keywords (Figure 5).

### 3.5. Cocitation by Cited Authors

**Figure 6:**
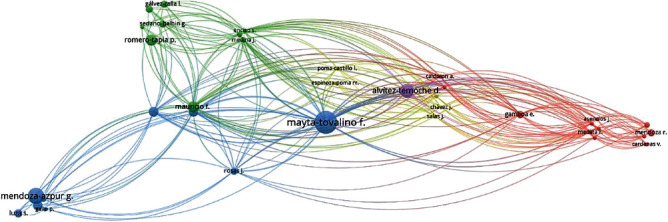
Cocitation by cited authors.

When evaluating the cocitation by cited authors, it was found that the author who had the greatest interrelation in the cocitation was Mayta-Tovalino F. who centralized the citations. The most representative authors who contributed to the cocitation were Alvitez-Temoche D. and Mendoza-Azpur G. (Figure 6).

### 3.6. Scholarly Output by Type of Collaboration

It was found that Peruvian dentistry publications in the period 2011-2020 mostly had 209 manuscripts (51.0%) of international collaboration with only 32 manuscripts (7.8%) that did not have any type of collaboration, and these publications of individual authorship had the lowest average citation per publication of 0.4 (Table 1).

### 3.7. Scholarly Output of Peruvian Dental Schools according to the Source

Concerning the journals that condensed the largest number of Peruvian publications on dentistry during 2011-2020, it was found that the *Chilean Journal of Oral Research* had the largest number of manuscripts (70) and brought together the largest number of Peruvian authors (139); however, this journal has a low average number of citations per publication with only 0.5 citations. The *Journal of Oral Medicine, Oral Pathology, and Oral Surgery* had only 11 publications, with an average of 11.2 citations per publication. This indicates that the quantity of publications does not guarantee adequate citation (Table 2).

### 3.8. Scholarly Output of Peruvian Dental Schools by CiteScore Quartile Journal

When evaluating the national scientific publications in dentistry according to the CiteScore, it was found that most of the publications (145) from Peru were published in Q4 journals, although 90 manuscripts were published in Q1 journals. The results of this analysis showed that there was an evident increase in the number of publications from 2018, 2019, and 2020 (Table 3).

### 3.9. Top 10 Universities of the Scholarly Output of Peruvian Dental Schools

When evaluating the top 10 of Peruvian universities with the highest scientific publication in the period 2011-2020, it was found that the Universidad Peruana Cayetano Heredia had the highest number with 105 manuscripts and the highest average number of citations per publication of 7.4 citations per article. The Southern Scientific University was found with 98 manuscripts and an average of 3.1 citations per publication. The public institution Universidad Nacional Mayor de San Marcos had 82 scientific publications and an average of 1.8 citations per manuscript. Finally, only three public universities were shown to be in the top 10 of institutions with the highest scientific productivity (Table 4).

### 3.10. Top 10 Authors of the Scholarly Output of Peruvian Dental Schools according to Sex

When analyzing the main bibliometric indicators according to the sex disparity of Peruvian researchers, it was found that in males, the most productive authors were Arriola-Guillen L. and Mayta-Tovalino F. with 38 and 31 manuscripts, respectively; both authors had the highest *h*-index, which was 6. Meanwhile, in relation to the female sex, the authors Guerrero María E. and Guevara-Canales J. were found to be the most productive with 23 and 15 manuscripts, respectively. However, the female authors' results were lower than those of the male authors (Table 5).

### 3.11. Top 10 Topic Clusters of Peruvian Dental Schools

It was identified that within the top 10 topic clusters of Peruvian dental publications during 2010-2020, the most investigated topics were as follows: caries, oral health, and mouth hygiene; Scotchbond, Clearfil SE Bond, and dentin; and smile, incisors, and orthodontists with 13, 10, and 10 manuscripts, respectively (Table 6).

### 3.12. Publications of Peruvian Dental Schools by Subject Area

When evaluating the scientific publications in Peruvian dentistry according to the subject area, it was found that the highest number of manuscripts (285) was in the subcategory “general dentistry,” with an average citation per publication of 4.1 citations per publication. Followed by the subcategory “oral surgery,” which encompasses 74 manuscripts with an average citation of 4.4 citations, and the subcategory “orthodontics,” which had 52 manuscripts with an average of 3.8 citations per publication (Table 7).

## 4. Discussion

The implementation of University Law 30220 in Peru has made a fundamental and necessary change to improve research in higher education. Before its implementation, many teachers were not involved in scientific research, making it difficult for students to produce scientific research. ^1-3^ Even the support of the thesis for the bachelor's degrees began to be required, which is a great step towards the scientific development of the country. These requirements have been better executed in certain institutions [9–12], and we can see it in the top 10 of Peruvian universities with the highest scientific publication, where we find that the Universidad Peruana Cayetano Heredia leads with 105 manuscripts and with an average of 7.4 in citations per publication. It was also shown that only three public Peruvian universities are in the top 10, and it can be inferred that to improve scientific publications, the correct administration of funds is necessary at the institutional level, as well as a better curriculum, more access to equipped laboratories, and hiring trained teachers.

Today, clinical practice and dental research cover various specialties, such as implantology, orthodontics, pediatric dentistry, restoration, and rehabilitation. As a result, scientific research in the dental field has increased in different lines of research. However, many researchers specialize in development in specific areas, often making collaborative work difficult. This can be evidenced in the coauthorship indices where four large clusters were led by three male researchers: Arriola-Guillen L., Mayta-Tovalino F., and Mendoza-Azpur G., and a female researcher: Guerrero María E. Similarly, in the cocitation index, the author with the highest cocitation interrelation was Mayta-Tovalino F. Therefore, multidisciplinary work should be developed to enrich scientific research.

In this bibliometric study, researchers from different licensed Peruvian universities were considered to evaluate the trend of scientific publications. The present investigation was not extended to public hospitals because these bibliometric parameters are not institutional indicators that allow the measurement of the work of clinical dentists in public health centers. In this way, the evaluation of scientific publications with bibliometric studies [13–20], such as this study, can improve the productivity of researchers.

The publication of original articles by male researchers has increased remarkably and constantly, and a maximum peak was evident in 2019. Meanwhile, there is no evidence of sustainable growth concerning female researchers. It is true that there are fewer female researchers; however, this does not happen in dentistry. The percentage of females is higher than that of males in the dentistry field, and their low publication in research could be explained by the fact that they tend to lean more towards clinical practice [6, 17–20].

The growing importance of women in scientific society should be emphasized, since women also publish research at a rate proportional to the number of men participating in each specialty. It is also important for women to be more active among themselves. This can involve the whole society so that there is a clear leadership equally between genders [21–23].

The main limitation is that Peru is a developing country, and there is a large research gap and multiple difficulties in accessing funds, equipped laboratories, and internship opportunities, and the vocation and interest to generate more knowledge stand out among researchers who have continued to publish steadily year after year. Here, we show evidence of collaborative work with other neighboring countries in Latin America, which has been of vital importance. We highlight Brazil as the main country that collaborates with Peruvian authors.

The main strength of this bibliometric study was to make a comparative bibliometric analysis at the gender level, also evaluating the academic production of dental schools at institutional levels. Finally, our results allow us to recommend that more complementary research be carried out by analyzing other databases like Web of Science and SciELO Citation Index, with the aim of determining the total production and impact of scientific dental production in Peru.

## 5. Conclusions

Peruvian national dental publication in the last 10 years was sustained mainly by male dentists, which invites reflection to reduce future gaps generated by the gender disparity of Peruvian researchers.

## Figures and Tables

**Table 1:** Scholarly output by amount of international, national, and institutional collaboration (2011-2020).

Metric	%	Scholarly output	Citations	Citations per publication	Field-Weighted Citation Impact
International collaboration	51.0	209	1454	7.0	0.94
Only national collaboration	24.6	101	143	1.4	0.33
Only institutional collaboration	16.6	68	125	1.8	0.61
Single authorship (no collaboration)	7.8	32	12	0.4	0.28

**Table 2:** Scholarly output of Peruvian dentistry schools in Scopus according to source (2011-2020).

Scopus source	Publications	Authors	Citations per publication	Source-Normalized Impact per Paper (SNIP)	CiteScore 2020	SCImago Journal Rank (SJR)
Journal of Oral Research	70	139	0.5	0.152	0.4	0.127
Revista Cubana de Estomatologia	31	70	0.2	0.141	0.2	0.124
American Journal of Orthodontics and Dentofacial Orthopedics	23	35	2.7	1.725	3.6	1.183
Revista Española de Cirugia Oral y Maxilofacial	17	35	1.2	0.163	0.3	0.126
Journal of Contemporary Dental Practice	15	54	2.1	0.607	1.3	0.3
International Journal of Dentistry	12	55	2.5	1.232	2.8	0.61
Medicina Oral, Patologia Oral y Cirugia Bucal	11	48	11.2	1.417	3.2	0.644
Journal of Clinical and Experimental Dentistry	9	41	6.2	1.119	2.5	0.481
Avances en Odontoestomatologia	8	18	0.1	0.171	0.2	0.119
Dental Press Journal of Orthodontics	8	17	3.3	1.362	2.1	0.57

**Table 3:** Scholarly output of Peruvian dentistry schools by CiteScore quartile journal (2011-2020).

CiteScore quartile	2011	2012	2013	2014	2015	2016	2017	2018	2019	2020	Total
Q1 (top 25%)	3	1	5	8	6	8	13	12	20	14	90
Q2 (top 26%-50%)	5	8	5	4	4	5	10	9	16	18	84
Q3 (top 51%-75%)	4	2	2	2	4	4	2	6	15	35	76
Q4 (top 76%-100%)	0	1	2	5	2	9	11	19	36	60	145
Total	12	12	14	19	16	26	36	46	87	127	395

**Table 4:** Top 10 universities of scholarly output of Peruvian dentistry schools in Scopus (2011-2020).

Universities	Scholarly output	Citations	Authors	Citations per publication	Field-Weighted Citation Impact
Universidad Peruana Cayetano Heredia	105	775	95	7.4	0.87
Universidad Científica del Sur	98	300	72	3.1	0.69
Universidad Nacional Mayor de San Marcos	82	151	98	1.8	0.49
Universidad de San Martín de Porres	55	331	59	6	0.55
Universidad Nacional Federico Villarreal	26	38	27	1.5	0.56
Universidad Peruana de Ciencias Aplicadas	21	91	38	4.3	0.62
Universidad Privada Antenor Orrego	21	34	14	1.6	0.24
Universidad Señor de Sipán	16	9	16	0.6	0.09
Universidad Nacional de Trujillo	15	10	14	0.7	0.08
Universidad Privada San Juan Bautista	11	29	24	2.6	0.95

**Table 5:** Top 10 authors of scholarly output of Peruvian dentistry schools in Scopus according to gender (2011-2020).

Gender	Name	Scholarly output	Most recent publication	Citations	Citations per publication	*h*-index
Male	Arriola-Guillén, Luis	38	2020	121	3.2	6
	Mayta-Tovalino, Frank	31	2020	50	1.6	6
	Aliaga-Del Castillo, Aron	22	2020	54	2.5	5
	Rodríguez-Càrdenas, Yalil	21	2020	57	2.7	4
	Mendoza-Azpur, Gerardo	20	2020	120	6	6
	Morales-Vadillo, Rafael	19	2020	74	3.9	6
	Ruíz-Mora, Gustavo Armando	18	2020	50	2.8	4
	Alvitez-Temoche, Daniel	18	2020	21	1.2	3
	Carruitero, Marcos J.	15	2020	29	1.9	3
	Bernabé, Eduardo	13	2020	108	8.3	50

Female	Guerrero, Maria Eugenia	23	2020	129	5.6	10
	Guevara-Canales, Janet-Ofelia	15	2020	74	4.9	6
	Delgado-Angulo, Elsa Karina	11	2020	97	8.8	9
	Casas-Apayco, Leslie	9	2020	57	6.3	5
	Chávez-Rimache, Lesly	9	2020	0	0	1
	Diaz, Karla	7	2019	175	25	5
	Malpartida-Carrillo, Violeta	7	2020	8	1.1	2
	Wang, Linda	6	2016	55	9.2	17
	Vergara-Buenaventura, Aandrea	6	2020	21	3.5	2
	Sacsaquispe-Contreras, Sonia	6	2019	64	10.7	6

**Table 6:** Top 10 topic clusters of Peruvian dentistry schools in Scopus (2011-2020).

Topic	Scholarly output	Publication share (%)	Field-Weighted Citation Impact
Caries; oral health; mouth hygiene	13	0.41	0.77
Scotchbond; Clearfil SE bond; dentin	10	0.41	0.75
Smile; incisor; orthodontists	10	0.75	0.16
Incisor; orthodontics; impacted tooth	9	1.03	1.54
Malocclusion; dental esthetics; orthodontics	8	0.8	0.75
Open bite; overbite; malocclusion	8	2.27	0.57
Pediatric dentistry; dentists; COVID-19	8	0.98	1.18
Dental pulp cavity; endodontics; E. faecalis	7	0.44	0.55
Alveolar bone; maxilla; inlays	6	0.37	1.08
Craniometry; hyoid bone; malocclusion	6	0.53	0.47

**Table 7:** Publications of Peruvian dentistry schools in Scopus by subject area (2011-2020).

Subcategory	Scholarly output	Citations	Authors	Citations per publication	Field-Weighted Citation Impact
General dentistry	285	1182	801	4.1	0.63
Oral surgery	74	323	240	4.4	0.63
Orthodontics	52	199	83	3.8	1.04
Periodontics	17	164	92	9.6	0.9
Dentistry (miscellaneous)	6	20	27	3.3	0.33
Dental hygiene	1	6	6	6	1.78

## Data Availability

The data that support the findings of this study are available from the corresponding author upon reasonable request.
